# Chronic kidney disease as a catalyst for cerebral microbleeds: understanding the underlying mechanisms and treatment approaches

**DOI:** 10.3389/fmed.2025.1578666

**Published:** 2025-06-25

**Authors:** Jia Yang, Xuezhi Chen, Xianming Cao, Hui Yang, Peiwen Liu, Xiaoping Yin, Xiaorong Zhang, Zhiying Chen

**Affiliations:** ^1^Department of Neurology, Affiliated Hospital of Jiujiang University, Jiujiang, China; ^2^Jiujiang Clinical Precision Medicine Research Center, Jiujiang, China; ^3^Department of Pathology, Affiliated Hospital of Jiujiang University, Jiujiang, China; ^4^Jiangxi Provincial Key Laboratory of Cell Precision Therapy, School of Basic Medical Sciences, Jiujiang University, Jiujiang, China

**Keywords:** cerebral microbleeds, chronic kidney disease, small vessel disease, amyloid, stroke

## Abstract

Cerebral microbleeds (CMBs) are tiny deposits of blood degradation products in the brain that appear as small, low-signal lesions on magnetic resonance imaging paramagnetic susceptibility sequences. They are common forms of the cerebral small-vessel diseases and are thought to be associated with serious consequences such as cognitive decline and increased risk of stroke. Chronic kidney disease (CKD) is a chronic disease in which renal impairment lasts for more than 3 months and is often accompanied by pathophysiologic changes such as inflammation, abnormal vascular endothelial function, and increased oxidative stress. It has been found that chronic renal insufficiency can induce the onset, development, and aggravation of cognitive impairment of CMBs, which may be caused by hypertension, inflammation and immune response, vascular lesions, blood–brain barrier damage, vitamin D deficiency, and so on. Therefore, it is essential to study the mechanism of cerebral microbleeds induced by chronic kidney disease to prevent the occurrence, development, treatment, and prognosis of stroke and related events in patients in the future. This article summarizes the definition, epidemiological investigation, pathophysiological mechanism, correlation, and treatment status of CMBs and CKD.

## 1 Introduction

Cerebral microbleeds (CMBs) are tiny deposits of blood degradation products in the brain, formed as small foci after lesions or damage to small blood vessels in the brain (1, 2). They are a type of cerebral small vessel disease. CMBs can cause clinical symptoms corresponding to cerebral hemorrhage, including stroke-like symptoms and cognitive deficits (3). However, they are not the same as a typical brain hemorrhage. In past studies, CMBs have often been used as biomarkers of stroke risk (4).

We learned that the pathogenesis of CMBs primarily involves erythrocyte leakage, evidenced by imaging manifestations and pathology. Pathologically, this suggests that they might be caused by erythrocyte leakage, as most CMBs correspond to ferritin-containing macrophages in neighboring vascular lesions (5). Ferritin-containing macrophages are specialized immune cells that store intracellular iron within ferritin, thereby preventing iron-mediated generation of reactive oxygen species. On T2-weighted Magnetic Resonance Imaging (MRI) images, it appears as a round or oval signal cavity with uniform low signal, typically 2–10 mm in diameter (6). However, some structures or lesions show imaging similar to CMBs, such as calcium and iron deposits in the bilateral basal ganglia, blood flow voids in the molluscum contagiosum vessels, and volume artifacts in parts of the skeleton in the temporal and frontal lobes (1). However, the iron-containing heme deposits that comprise the CMBs are superparamagnetic (1, 7). When an MRI was performed, the magnetic field near CMBs could become inhomogeneous, showing the signal loss in the focal areas of their brain parenchyma. These can be identified with others. However, the correlation between low signal on imaging and ferritin deposits is not absolute, and the correlation needs to be studied in depth. Lesions of small blood vessels in the brain can be easily overlooked and missed because of the small size of the lesions and the insidious appearance of the symptoms. However, in recent years, with the popularization of magnetic resonance equipment and magnetic susceptibility sequences, the study of CMBs has become more and more in-depth.

Chronic kidney disease is defined as a glomerular filtration rate (GFR) of less than 60 mL/min per 1.73 m^2^ (GFR classification G3a-G5) or an albumin creatinine ratio (ACR) ≥ 30 mg/g [3 mg/mmol] and renal structural and functional impairment (1 or more markers of kidney injury) that lasted for more than 3 months, both GFR and ACR are important indicators of kidney function. GFR is usually calculated by estimating endogenous markers (8). According to the internationally recognized guidelines of the American Kidney Disease Foundation, chronic kidney disease is clinically classified into five stages, and the development of more than three stages is chronic kidney failure (9); it is the outcome of the continued progression of various chronic kidney diseases. Chronic Kidney Disease (CKD) is considered a risk factor for cerebrovascular disease (CVD) and a risk factor for stroke (10). Epidemiological studies have shown that chronic kidney disease currently affects 10–14% of the world’s population (11). Nevertheless, only 6% of the general population and 10% of those at risk are aware of their CKD status (12). According to statistics, CKD and its effects on CVD led to 2.6 million (95% UI: 2.4–2.8) deaths in 2017 (8); this suggests that the impact of CKD on CVD is gradually expanding, and it is crucial to study the mechanisms involved. Notably, the increasing prevalence of Chronic Kidney Disease (CKD) may constitute a novel risk for CMBs development. One study found a positive correlation between CMBs and serum creatinine levels in an established CKD mouse model (Spearman r = 0.37, *p* < 0.01) (13), suggesting that CKD has an inducing formation and promoting effect on CMBs. Cystatin C is considered a more sensitive kidney marker than conventional kidney markers (14); it can be used to estimate the size of the GFR and can be affected by factors such as obesity, thymic disease, hormones, and smoking (8). It was found that the proportion of patients with moderate to severe CMBs increased with increasing cystatin C levels (p for trend <0.01) (15). All these studies illustrate a link between CKD and CMBs, and CKD can induce the development of CMBs.
In this review, we reveal the potential link between chronic kidney insufficiency and CMBs, analyze the clinical features, prognosis, and therapeutic strategies of CMBs in patients with chronic kidney insufficiency, as well as provide an outlook on future research directions and clinical applications in this field. In particular, we comprehensively summarize the possible mechanisms by which CMBs are induced in chronic kidney disease, including vascular factors, metabolic factors, hypertension, blood pressure variability, and inflammatory and oxidative stress factors, and collect a variety of epidemiological investigations as well as analyze the potential mechanisms in animal models for a variety of clinical conditions. We also highlight the latest study in which the extent of cerebral hemorrhage was assessed by quantifying the degree of vascular calcification through estimated glomerular filtration rate (eGFR), which provides new ideas and methods for determining the relationship between CKD and CMBs. In conclusion, the analysis of published data from preclinical and clinical studies provides substantial support for possible mechanisms of CKD-induced CMBs.

## 2 Methods

This narrative review synthesises current literature, relying on the methodology outlined in the following subsections.

### 2.1 Search strategy

The review utilized PubMed, CNKI, Cochrane, Web of Science, BMJ, and Wan fang digital journal full-text databases, and references from relevant articles, with various combinations of the search terms such as “chronic kidney disease,” “cerebral microbleeds,” “cerebral microbleed,” “cerebral amyloid angiopathy,” “small vessel disease,” “stroke”. Boolean operators (e.g., AND, OR) were used to combine search terms with synonyms and refine the search results.

### 2.2 Types of literature and time frame

Articles solely reported in the form of abstracts or meeting reports were excluded. Studies published between 2000 and 2025 were included to ensure the selection of the most relevant and up-to-date research. In order to maintain rigor and greater clarity in the content of the review, only English-language publications were considered.

### 2.3 Data and reference management

Endnote literature management software was used to systematically document and organize detailed records of the search results, screening processes, and references.

## 3 Epidemiologic study of chronic kidney disease and cerebral microbleeds

### 3.1 A survey of the incidence of cerebral microbleeds

The prevalence of CMBs has been documented in multinational studies; in 2006, the prevalence of CMBs in the United Kingdom was found to be around 5% in “healthy people” by MRI in 1,411 participants (16); in recent years, the prevalence of CMBs is around 5% in “healthy people” by large-scale studies in community-based populations, e.g., the Framingham Study The prevalence of CMBs was 10.8% in the Framingham study (17), 15.3% in the Rotterdam study (18), and 11.1% in the Reykjavik study (19). In China, the prevalence of CMBs was found to be 10.6% in the Beijing Shunyi community-based population study (20). Differences arise due to factors such as assay methodology or subject population.

Different age groups will have different prevalences of CMBs. In earlier studies, the prevalence of CMBs was 6.5% in people aged 45–50 and 35.7% in people aged 80 and above (18). In recent years, it has been shown that the prevalence of CMBs is 11% between the ages of 60–69 years, 22% between the ages of 70–79 years, and 39% at the age of 80 years and above (21). These data imply that the prevalence of CMBs is positively correlated with age, and the prevalence of CMBs is gradually increasing in all age groups. The prevalence of CMBs in patients with ischemic stroke is 34% (16), and ischemic stroke increases with the increasing burden of CMBs (22). In patients with non-traumatic cerebral hemorrhage (ICH), it is 60% (16), implying that the prevalence varies among patients with different cerebrovascular diseases.

### 3.2 A survey of the incidence of chronic kidney disease

Chronic kidney disease (CKD) is not evident in the early stage, so there may be bias in the prevalence statistics. A cross-sectional survey revealed that the prevalence of chronic kidney disease (CKD) among adults in China was 10.8%, with variations in prevalence across different regions (23). A 2017 survey showed that the global prevalence of CKD was 9.1%, and of the 697.5 million cases of CKD patients, there were more than 10 million cases of CKD in some developed and developing countries (24). CKD needs to be emphasized in both developed and developing countries.

### 3.3 A study on the correlation between chronic kidney disease and cerebral microbleeds

Epidemiologic studies have focused on exploring the prevalence of CMBs in patients with CKD. A survey conducted by Toyoda et al. found that the prevalence of CMBs was significantly higher in patients with CKD than in the general population, especially in patients with end-stage kidney disease (25). The study also pointed out that the degree of kidney function impairment in CKD patients was positively correlated with the prevalence of CMBs, suggesting that the decline in kidney function may be related to CMBs. Another study by Nagai also found that the prevalence of CMBs was significantly higher in patients with CKD than in non-CKD patients, especially in patients with more severe CKD (26). In addition, this study found that the prevalence of CMBs was further increased in CKD patients with comorbidities such as diabetes mellitus and hypertension, suggesting that a combination of factors may influence the occurrence of CMBs in CKD patients. In another cross-sectional study using T2-weighted sequences of brain MRI in CKD patients and normal subjects, CMBs were detected in 25.6% of CKD patients but not in controls (27). Although these cross-sectional studies suggest a potential link between CKD and CMBs, longitudinal studies are essential to determine temporal causality and to elaborate on the relationship between CKD-promoting CMBs.

Longitudinal studies have shown that impaired kidney function was a risk factor for CMBs in the deep or subcuratorial region (OR = 1.533, 95% CI, 1.111–2.114; *p* = 0.009), as well as for increased CMBs (OR = 2.577, 95% CI, 1.393–4.769; *p* = 0.003), independently of lobular-region CMBs (28). As mentioned earlier, one of the key indicators of kidney function assessment is the glomerular filtration rate (GFR). Several studies have shown that the Estimated Glomerular Filtration Rate (eGFR) is associated with the progression of CMBs (29, 30). The risk of CMBs was significantly higher as the level of eGFR decreased (30). This suggests an independent correlation between eGFR and the progression of CMBs in the presence of impaired kidney function, reinforcing the link between chronic kidney insufficiency and CMBs. Thus, we clarified that CKD is an independent risk factor for the development of CMBs. Further studies are needed to deeply explore the relationship between CKD and CMBs and provide a more reliable basis for developing effective preventive and therapeutic strategies.

Based on these epidemiologic studies and longitudinal studies, we next further investigated the pathophysiologic mechanisms linking CKD to CMBs to provide more effective strategies for the prevention and treatment of cerebrovascular events.

## 4 Pathophysiologic mechanisms of cerebral microbleeds induced by chronic kidney disease

### 4.1 Pathogenesis of cerebral microbleeds in chronic kidney disease

A study observed a 67-year-old female patient with end-stage kidney disease for 2 years, during which time increasing foci of CMBs were found, accompanied by cognitive decline (31). It has been shown that the association between cystatin C and CMBs in elderly subjects has been analyzed, and the proportion of subjects with CMBs was lower in the group with lower cystatin C levels, especially in subepithelial microbleeds (32). As cystatin C is an accurate indicator of kidney function, this study showed that CMBs are more likely to occur when kidney function is low (15). These studies have demonstrated significant CMBs in patients with chronic kidney insufficiency. Therefore, based on the anatomical findings that both the kidney and the brain have a constant and stable high blood flow in a low vascular resistance system (33), we hypothesized that CMBs occur due to the vasculature being affected in patients with CKD. The mechanism may be calcification of vascular damage and impairment of the blood–brain barrier, while a new mechanism of erythrophagocytic action has been demonstrated in cell culture systems. In a prospective study (34), which included 431 patients with no history of cerebrovascular disease, it was demonstrated that each 1 SD increase in the levels of various inflammatory markers was significantly associated with the presence of CMBs, suggesting that inflammation plays an important role in the progression of CKD (35). Therefore, it is hypothesized that CKD can induce inflammation and promote the occurrence of CMBs. Currently, the related mechanisms of chronic kidney insufficiency inducing CMBs are (1) vasculopathy: the accumulation of calcium and phosphorus in the advanced stage of CKD can easily cause vascular calcification, which decreases the compliance of renal and cerebral small blood vessels, and then triggers CMBs; (2) high blood pressure. Most CKD patients have different degrees of hypertension due to water and sodium retention and elevated renin-angiotensin, etc., and small cerebral arteries are easily affected by hypertension, which may lead to CMBs; (3) blood pressure variability. A study (36) found that CKD is correlated with increased blood pressure variability, which is also considered a risk factor for CMBs; (4) Erythrophagy. One study *in vitro* cell culture found that brain endothelial cells exposed to oxidative stress can express erythrophagocytic phenotype in erythrocytes (37); (5) Inflammation and immune response. A study (38) established a mouse model of nephritis and found that it could induce the development of CMBs; (6) Blood–brain barrier lesions. Patients with CKD are prone to disruption of the blood–brain barrier due to inflammatory response, elevated blood pressure, and oxidative stress, leading to the development of CMBs. (7) Vitamin D deficiency: CKD patients are prone to vitamin D deficiency during disease progression, which triggers hypertension, inflammation, oxidative stress, etc., and promotes the occurrence of CMBs.

### 4.2 Mechanisms associated with cerebral microbleeds induced by chronic kidney disease

**Figure 1:**
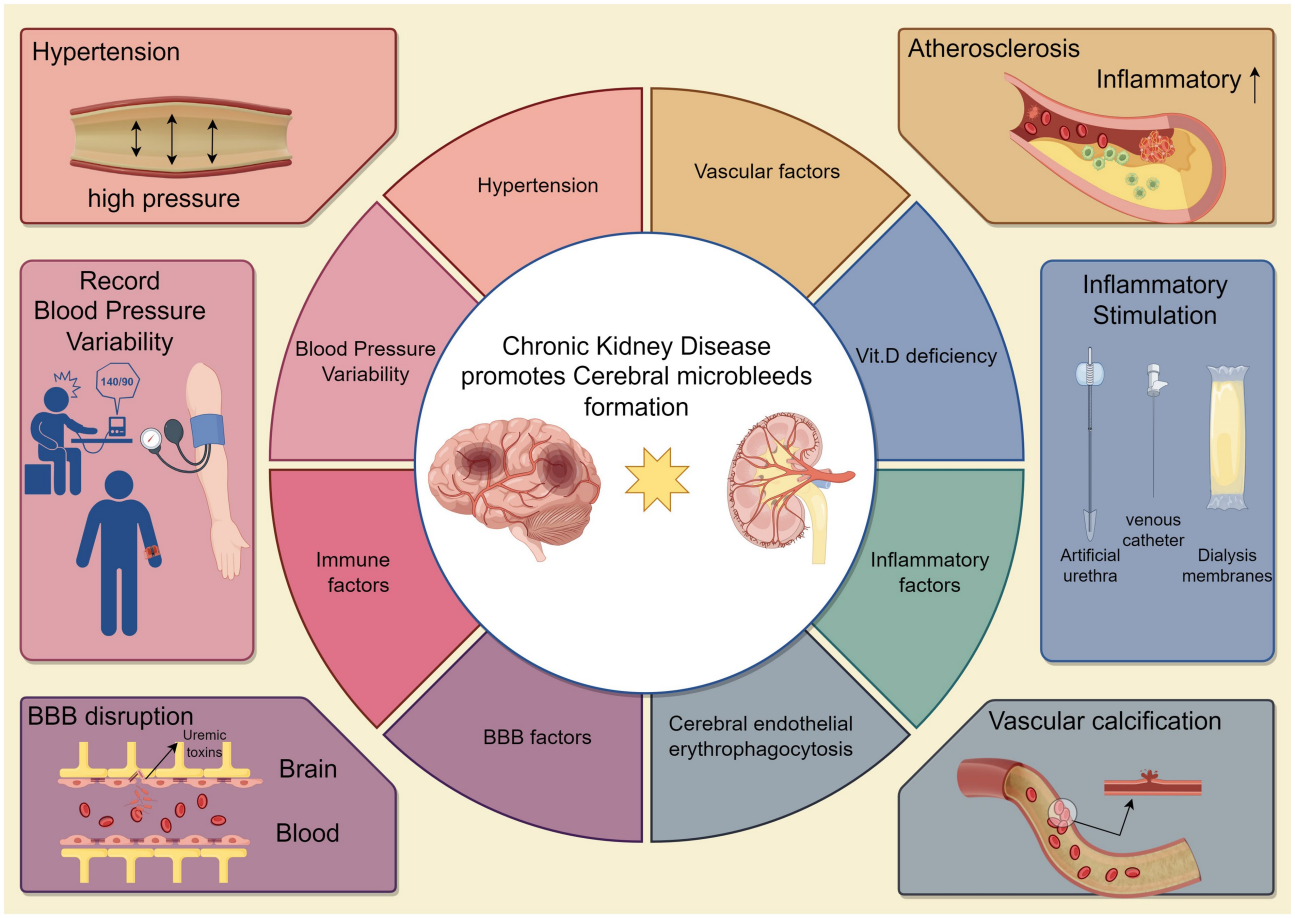
Chronic kidney disease partially induces macroscopic mechanisms associated with cerebral microhemorrhages. Chronic kidney disease induces cerebral microhemorrhage, which can be inferred from several macroscopic indicators such as hypertension, atherosclerosis, blood pressure variability, inflammatory and immune factors, blood–brain barrier, and vascular calcification. Short blood vessels linked to the brain during the development of CKD become strain vessels, and prolonged exposure to high pressure is prone to cause damage to the microvasculature. In addition, CKD patients with vitamin D deficiency have increased permeability of the blood–brain barrier due to exogenous infections or inflammatory reactions triggered by accumulation of toxins in the body as a result of improper therapeutic maneuvers. The arterial vasculature is prone to hemodynamic changes and atherosclerosis in response to inflammation and oxidative stress. These figurative macroscopic representations can provide support for further in-depth exploration of specific microscopic mechanisms (By Figdraw.).

**Figure 2:**
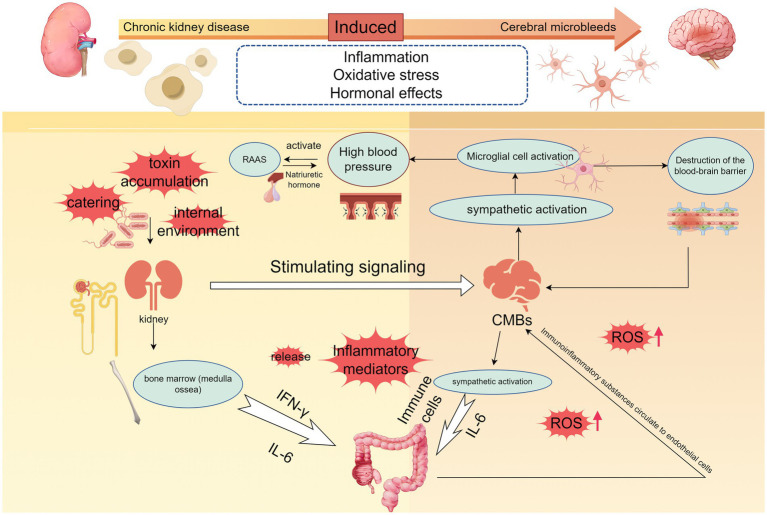
The microscopic mechanisms of chronic kidney disease-induced cerebral microbleeds are locally relevant. The development of hypertension is accompanied by the development of chronic kidney disease, and when dietary, environmental, or other stimulus signals such as accumulation of uremic toxins are transmitted to the brain to activate the sympathetic nerves, the persistent activation of microglia and neuroinflammation occurring promotes the development of hypertension, and disruption of the blood–brain barrier. Elevated blood pressure also activates the RAAS system, and hyperperfusion promotes cerebrovascular damage. Immune cells are released into the gut and kidneys, destroying gut flora and PH, and immune substances end up in the renal circulation. When vitamin D deficiency, oxidative stress will further promote the release of inflammatory factors, part of the inflammatory response can also be achieved through the NF-κB signaling pathway, a large number of inflammatory factors to promote increased permeability of the brain barrier, damage to the vascular endothelium, accelerated the formation of CMBs (By Figdraw.).

In terms of mechanisms, we discussed macro (Figure 1) and micro factors (Figure 2) in terms of vascular factors, inflammatory and immune factors, blood–brain barrier factors, and metabolic factors, thus enabling a more comprehensive understanding of how CKD effectively contributes to the development of CMBs at both macro and micro levels.

#### 4.2.1 Effects of hypertension on CMBs in CKD

Epidemiologic investigations have found that the prevalence of CMBs is significantly higher in CKD patients with a history of hypertension than in CKD patients without a history of hypertension, and the number of CMBs increases with age (39). Therefore, it is necessary to investigate whether there is an association between the induction of CMBs in hypertensive CKD patients.

A study found that in 142 patients with acute intracerebral hemorrhage (ICH), CKD was positively correlated with a history of chronic hypertension (*p* = 0.046) and the prevalence of MRI overall and deep CMBs (*p* = 0.001, *p* = 0.002) (40). This correlation increased as the prevalence of CMBs increased with more severe chronic kidney disease stages. This correlation was the same in another study of an older population (41). In the multicenter longitudinal cohort study of CRIC, the association between CKD and hypertension at only one time point in previous observational studies was reinforced (42). These studies link hypertension, chronic kidney disease, and CMBs, suggesting a strong association between hypertensive vasculopathy in patients with chronic kidney disease and the occurrence of CMBs. An in-depth examination of hypertension as a strong causative agent of CMBs in patients with chronic kidney disease may be explained through the vascular pressure dimension.

In physiology, we know that the brain and kidneys have high blood flow and local autoregulation; the pathology of CMBs is similar to that of CKD. In the “vascular hypothesis,” it is recognized that branches of the anterior, middle, and posterior cerebral arteries that penetrate brain tissue, the central retinal artery of the eye, the coronary arteries of the heart, and the small proximal medullary arteries of the kidneys are referred to as strain vessels, and that these short blood vessels are generated directly from high-pressure arteries that are exposed to high pressures over short distances, providing large pressure gradients (43). It is this anatomy that creates the conditions for microvascular injury. Arterial stiffness is one of the factors leading to microvascular injury when exposed to factors that increase pressure or damage strain vessels (39). Following microvascular injury, autoregulation is lost and sustained hypertension continues to exacerbate atherosclerosis, creating a vicious cycle that perpetuates end-organ damage and leads to the formation of CMBs. Thus, hypertension-induced CMBs have become the classic damage pattern in chronic kidney disease.

In addition to some macrostructures and data at the clinical level, studies at the micro level are equally valuable. During the development of CKD, systemic blood flow regulation due to increased renal water and sodium retention or secretion of sodium-removing hormones also induces an increase in blood pressure (44). The sustained pressure and load on the cerebral vasculature caused by hypertension increase the tendency for vascular rupture. Hypertensive vascular injury occurs first in such strained vessels, which in turn leads to the development of hypertension (25, 43, 45).

It is well known that chronic kidney disease is characterized by dysregulated vitamin D and mineral metabolism (46). As glomerular filtration rate (eGFR) declines, levels of both the stored and active forms of vitamin D decrease (47). 25 (OH) D is the major circulating form of VD in the body and an independent inverse predictor of stage 2–5 chronic kidney disease, with synthesis largely dependent on the bioavailability of the cutaneous precursor cholecalciferol and on hepatic 25-hydroxylation (48, 49). Endocrine guidelines define vitamin D deficiency as 25 (OH)D < 20 ng/mL and vitamin insufficiency as 25 (OH) D between 21–29 ng/mL (50). 1,25(OH)_2_D_3_ deficiency occurs during the progression of CKD, and reduced renal 1-alpha-hydroxylase activity typically results in reduced 1,25(OH)_2_D_3_ and hypocalcemia (51, 52). In the relationship between vitamin D and cerebral small-vessel disease, a negative association between cerebral small-vessel disease and 25 (OH) D was found using a Mendelian randomization method, given the ability of 25(OH)D to be a target of intervention (53–55). On average, the odds of deep cerebral small-vessel disease increased by 1.28 for every 25 nmol/L decrease in 25(OH)D (56). The mechanism is thought to be related to hypertension. There is an inverse relationship between 25(OH)D levels and blood pressure (57), while genetically and observationally low 25 (OH)D levels are associated with elevated blood pressure (58). Thus, the risk of cardiovascular events, including hypertension, appears to be higher in patients with low circulating 25 (OH)D levels, as confirmed by relevant studies (59, 60).

Previous studies have also found that the interplay of multiple factors, including sympathetic, vascular, and renin-angiotensin systems, such as elevated blood pressure has implications for activation of the Renin-Angiotensin-Aldosterone System (RAAS) as well as regulation of vascular tone (61). It is possible that this is related to the role of epigenetic factors in the brain-gut-kidney axis (62). When dietary, environmental, and other pro-hypertensive and CKD-related stimuli are perceived by autonomic brain regions, the resulting signaling to the gut and bone marrow can drive sympathetic activation. Sympathetic activation in the brain triggers continued microglia activation and neuroinflammation, leading to increased blood pressure (62). This is followed by the activation of immune cells that are released into the gut and kidneys, activation of the gut microbiota, influx into the kidneys, and ultimately inflammation of the gut and kidneys. This novel perspective of a series of pathological events largely elucidates the process by which the brain and gut contribute to the development of hypertension and CKD. Therefore, in response to the brain-gut-axis hypothesis, inhibition of sympathetic activation with minocycline is effective in the treatment of such diseases (63).

#### 4.2.2 Effects of blood pressure variability on CMBs in CKD

Blood pressure variability (BPV) has an indelible role in stroke. Several studies have shown that blood pressure variability is an independent risk factor for stroke. And a 24-h ambulatory blood pressure study in patients with acute ischemic stroke found that the nocturnal systolic standard deviation and nocturnal diastolic standard deviation in the deep CMBs group were higher than those in the non-CMBs group (*p* < 0.05) (64). This suggests that BPV is more correlated with CMBs. Blood pressure variability (BPV) independently predicts the progression of CMBs in deep and subcurtain regions (36). And both long-term and short-term BP variability were found to be risk factors for CKD progression (65). In non-dialysis patients with intermediate to advanced CKD, increased BP variability was independently associated with an increased risk of hemorrhagic stroke (66), which links CKD to stroke and suggests that the presence of BPV in patients with CKD has a predisposing role in the development of CMBs.

In addition to looking for an association between the two in risk factor studies, through various studies on the mechanisms by which CMBs are induced by changes in blood pressure in patients with CKD, it has been suggested by meta-analysis that CMBs may be induced by atherosclerosis or impaired endothelial function (67). This has been confirmed in a study on the relationship between blood pressure variability and vascular function (68). In addition to this, factors such as inflammation, sympathetic activity, and regulation of NO levels also have an impact on blood pressure variability in study populations with reduced endothelial function (69, 70). This leads to the conclusion that blood pressure variability can influence the progression of CMBs in patients with CKD. However, this aspect of the study is not sufficiently well researched to determine whether there is an association or dissimilarity between this sympathetic activity, inflammation, and induced CMBs in hypertensive CKD patients, and more evidence is needed to strengthen this association in future studies.

#### 4.2.3 Effects of vascular factors on CMBs in CKD

It is because of the similar anatomy of CKD and CMBs that vascular factors have an important role in the promotion of CMBs by CKD.

##### 4.2.3.1 Atherosclerosis

Atherosclerosis is a multifocal immunoinflammatory disease (71). Atherosclerosis is said to occur when the endothelium of a blood vessel is disrupted and damaged, resulting in a gradual accumulation of plaque on the inner wall of the vessel and affecting hemodynamic changes (72). The aortic endothelium can be visualized by imaging and other methods, showing a macroscopic morphology. It has been found that widening of the subendothelial gap in patients with kidney injury is suggestive of endothelial cell dysfunction (73), one of the features of which is an endothelial phenotype that promotes thrombosis (74). Once atherosclerosis occurs at the specialized vascular structural connection between the kidney and the brain, hemodynamic changes occur. Inflammation may be an important substance linking atherosclerosis, chronic kidney disease, and CMBs. Detection of inflammation in potential intracranial atherosclerotic plaques by high-resolution magnetic resonance imaging effectively confirms this hypothesis (75). As the degree of carotid atherosclerosis increased, the mean number of points of CMBs also increased (*p* < 0.01) (76). The inflammatory environment creates the formation of atherosclerosis and promotes the development of CMBs, and the inflammatory environment is chronically present in patients with chronic kidney disease. Therefore, atherosclerosis has a potential role in promoting the development of CMBs by trending in the *in vivo* environment of CKD patients.

##### 4.2.3.2 Vascular calcification

Atherosclerosis triggers structural damage to the vasculature as well as vascular abnormalities that are thought to be caused by vascular calcification in this layer of the vessel wall (77, 78). Vascular calcification is characterized by damage and wear and tear of vascular smooth muscle cells and can be divided into calcification of the outer and inner membrane layers, whereas the predominant form of calcification in chronic kidney disease is endothelial calcification (79). Vascular calcification in CKD cannot be separated from the calcium and phosphate accumulation that characterizes the development of the disease itself. Prolonged exposure to calcium or phosphate makes CKD patients more likely to accumulate calcium and phosphate compared to non-chronic kidney disease patients or normal individuals. This has also been demonstrated in an *in vitro* culture model of human blood vessels (80). It is also related to other factors in addition to the characteristics of their own disease progression. Studies have shown that phosphate overload in CKD patients may be associated with downregulation of membrane-bound protein II (MBI) (80, 81) and altered osteogenic phenotype of vascular smooth muscle cells or upregulation of related genes (82). Calcium tends to be deposited in ectopic soft tissues that include the vascular system (80, 83). The reason may be that CKD patients are more deficient in endogenous calcium inhibitors than healthy individuals (33, 84–86). Thus, elevated calcium and phosphate in CKD patients contribute largely to vascular calcification (87).

Is there a correlation between cerebrovascular dysfunction and vascular calcification in CKD? The answer is yes. It has been shown in animal models that cerebrovascular dysfunction is caused by kidney injury from vascular calcification in CKD and that CKD increase CMBs burden (88). The reason for this is that vascular calcification reduces vascular compliance and disrupts the ability of small arteries to compensate for changes in blood pressure in the body circulation, and hemodynamic changes make it difficult for the brain to adapt to changes in blood pressure, resulting in small artery injury. Vascular smooth muscle damage and small artery damage are the vascular sources of microbleeds. Sustained small artery damage limits cerebral blood flow autoregulation, and hyperperfused blood flow ultimately contributes to the development of CMBs (89). Vascular calcification plays a role in this mechanism to suggest the extent of the occurrence of CMBs, and recent macroscopic metrics studies have pointed out that eGFR can rigorously quantify the extent of vascular calcification and predict cerebral hemorrhage by using intracranial calcification (IAC) as an intermediate biomarker (90, 91) (Table 1). It suggests that we can understand renal function by quantifying the degree of vascular calcification, furthermore, more visualize the mechanism of CKD-induced CMBs. In conclusion, the formation of vascular calcification in CKD has a certain promotion effect on the occurrence of CMBs, and quantifying the degree of vascular calcification and then adopting various therapeutic means may be able to delay the deterioration of renal function and prevent the occurrence of CMBs (Table 2).

**Table 1:** Risk factors for CKD-induced CMBs.

Relevant risk factors	Injury site and mechanism	Primary link	Literature reference
High blood pressure	Degeneration or damage to cerebral arteries	In relation to the stimulatory effects associated with pro-hypertension and CKD, elevated blood pressure in CKD patients leads to cerebral sympathetic activation of microglia with neuroinflammation, with consequent damage to small arteries, and hyperperfused blood flow triggering CMBs.	Yang et al. (62)
Blood pressure variability	The inability of blood vessels to maintain autoregulation	Due to the inability of cerebral blood vessels to maintain autoregulation over a wider range of blood pressures, the vascular endothelium is susceptible to damage in the presence of inflammation, neuromodulation, and other conditions that lead to changes in blood pressure, which can lead to CMBs.	Zhang et al. (69), Diaz et al. (145)
Calcification of arteries	vascular endothelial dysfunction	The development of CKD contributes to the formation of vascular smooth muscle wear and vascular calcification, and the risk of cerebral small-vessel disease is significantly elevated as intracranial calcification rises significantly with the severity of CKD staging.	Mazzacane et al. (90), Li et al. (91)
Inflammation response	Activation of the immune system, stimulation of the brain endothelium by inflammatory factors	A series of factors such as catheter and dialysis membrane infections or the presence of vitamin D deficiency, uremic toxins and other factors may occur during the development of CKD, leading to the release of inflammatory factors, triggering intestinal flora dysbiosis, accelerating the release of immune substances, and triggering cerebral microvascular damage in a long-term inflammatory environment.	Csiszar et al. (45), Tsuji et al. (110)
Blood–brain barrier disruption	Destruction of intracerebral barrier structures	Urotoxins produced by CKD patients damage the brain endothelium and the blood–brain barrier through oxidative stress and activation of microglia, thereby triggering CMBs.	Watanabe et al. (146)

**Table 2:** General strategies for controlling CKD, CMBs, and the development of CKD-induced CMBs (8, 41, 147–149).

General strategies to prevent CKD	General Strategies to Prevent CMBs	General strategies to prevent CKD-induced CMBs
Controlling smoking	Controlling smoking	Closely monitor the extent of vascular calcification in patients with CKD.
Controlling weight	Controlling antithrombotic drug therapy	Improvement of uremic toxins as well as intestinal imbalances in CKD patients.
Controlling blood sugar levels	Controlling blood sugar levels	Supplemental vitamin D levels were given to CKD patients.
Controlling of blood pressure	Controlling of blood pressure	Control of common risk factors in patients with CKD.
Monitoring of blood lipid levels	Monitoring of blood lipid levels	
Reducing proteinuria		

#### 4.2.4 Effects of inflammation and oxidative stress on CMBs in CKD

The correlation between inflammation and CMBs has been studied, and a chronic inflammatory environment favors microvascular injury (45). However, we found that the inflammatory response frequently occurs in patients with chronic kidney disease, which is multifactorial. Exogenous factors may be urethral tubes, central venous lines, dialysis membranes, etc., which can be avoided in good clinical practice (92). Endogenous factors include immune dysfunction, intestinal flora dysbiosis, and retention of uremic toxins (93), which may activate the immune system and lead to a systemic inflammatory response (Table 1). Therefore, we discuss the mechanism of inflammatory response on the formation of CMBs in CKD.

Oxidative stress underlies the development of chronic diseases, including CKD, and cellular senescence is marked by oxidative stress. CKD is considered to be a premature aging of the kidneys characterized by increased cellular senescence, a state of irreversible cell cycle arrest, and cessation of cell division (94). These changes are inseparable from the role of factors. With age, SASP factors secreted by senescent cells of the neurovascular unit can propagate senescence to neighboring cells (95). Senescent vascular cells produce more ROS, which are characterized by the production of more inflammatory cytokines and chemokines and altered synthesis of lipid mediators (96), thereby amplifying the damage to microvascular function and integrity.

In contrast, the occurrence of oxidative stress in the kidney has been implicated in sex hormones. Estrogen exerts a protective effect on the kidneys by reducing the production of reactive oxygen species, and this protective effect is also reflected in the correlation between the Chinese visceral adiposity index (CVAI) and CKD (97). Testosterone, on the other hand, increases oxidative stress associated with kidney injury by increasing the production of reactive oxygen species and inhibiting antioxidant enzymes (98, 99). Estrogens also inhibit TGF-β1, TNF-*α*, and T-cell-induced apoptosis (100), whereas testosterone hormones are prone to triggering apoptotic effects in renal cells (101). Thus, sex hormones, especially testosterone, cause kidney injury during oxidative stress and promote the development of CKD. The existence of opposite effects of the two sex hormones on the kidney suggests that oxidative stress can act differently on the kidney with different hormones. Future studies utilizing the mechanism of action of these kidney-protective hormones may suggest new therapeutic strategies. Meanwhile, it was demonstrated in a recent study that the Chinese Visceral Adiposity Index (CVAI) was linearly associated with new-onset stroke in middle-aged and older Chinese adults (102). This gives us a hint whether reducing CVAI in future studies could reduce the probability of stroke in CKD patients. However, it has also been suggested that CKD is a neutrophilic state, and salt itself tends to stimulate the cerebral cortex to produce reactive oxygen species of inflammatory cytokines (103), and that too much sodium can exacerbate the inflammatory phenomenon of CKD and cause corresponding damage.

Uremic toxins are considered important substances involved in inflammatory and immune responses. Uremic toxins are predominantly found in advanced stages of chronic kidney disease (104) and can be categorized as endogenous, exogenous, and microbial sources (105). These toxins may be due to the entry of residual urea and other waste products from the body of CKD patients into the lumen of the gastrointestinal tract, which affects the pH of the lumen and leads to an imbalance in the composition of the intestinal microbiota, such as indolephenol sulfate, p-formyl sulfate, and trimethylamine N-oxides (TMAO), as well as in their metabolic capacity (106–109).

The gut-kidney axis, which has been studied in recent years, links the two types of organs and can be subdivided into metabolism-dependent pathways and immune pathways (110). In the metabolic-dependent pathway, dysbiosis leads to the cumulative release of uremic toxins. In the immune pathway there is activation of a large number of immunogenic substances, such as macrophages, T cells and other resident immune system cells, which disrupts intestinal permeability and promotes the production of inflammatory mediators, such as IL-6, gamma interferon (IFN-*γ*) and tumor necrosis factor-*α* (TNF-α) (111, 112). It also activates the transcription factor NF-κB, which increases the expression of inflammation-related genes and exacerbates the immune-inflammatory response (Table 1). It also activates the transcription factor NF-κB, which increases the expression of inflammation-related genes and exacerbates the immune-inflammatory response. The products of these metabolite imbalances eventually flow into the kidneys to achieve circulation, leading to the development of chronic inflammation in the kidneys.

In addition, vitamin D deficiency may be involved in the development of inflammation in patients with CKD. A 10-unit increase in serum 1,25(OH)2D or 25(OH)D is associated with a decrease in renal inflammatory response (113). In healthy control experiments, vitamin D-deficient subjects had higher serum concentrations of inflammatory factors and higher CCL-2, NF-κB2, and NF-κB3 gene expression than vitamin D-sufficient subjects (114). The low vitamin D levels suggest that CKD patients may be able to schedule vitamin D supplementation to replace deficiencies and prevent the development of other diseases. However, this is only an ideal state, and no relevant experimental clinical studies have been found for the time being, and further expansion of the validation range is needed in the future (Table 2).

Therefore, we believe that oxidative stress occurs in CKD in a multifaceted manner, but its pro-inflammatory and induced process of CMBs is the focus of our discussion. Elevation of these inflammatory mediators is also present in the occurrence of CMBs (34). To further validate this, a study modeling inflammatory mice found that inflammatory mice were significantly more positive for CMBs than controls (96), and aging also exacerbates this process. This not only reinforces the relationship between oxidative stress and inflammation, but also validates the correlation between CMBs and inflammation. The release of ROS directly activates immune cells, prompting them to release more inflammatory mediators, which can be more clearly demonstrated in uremic toxins and in atherosclerosis (74, 115, 116). The release of these proinflammatory factors, such as C-reactive proteins, cytokine IL-6, and other inflammatory compounds (117–120), affects vascular cells and promotes cerebral microvascular injury, leading to the development of CMBs.

In summary, inflammation may be a potential trigger for CKD in promoting the development of CMBs. Controlling the inflammatory process in CKD may be helpful in mitigating the development of CMBs. Use of probiotics, prebiotics, and a low-protein diet could be a new way to control disease progression, according to the gut-kidney axis hypothesis (121). However, a large number of clinical trials are still needed for further validation.

#### 4.2.5 Effects of blood–brain barrier on CMBs in CKD

Blood–brain barrier damage is accompanied by loss of various physiological functions and is an important pathological feature of many brain diseases. The blood–brain barrier (BBB) consists of the basal lamina, endothelial cells, astrocytes, and pericytes. It has been found that the blood–brain barrier becomes dysfunctional during normal brain aging in both humans and mice (122). Impairment of the blood–brain barrier is predictive of cerebral hemorrhage to some extent (123). Therefore, improved assessment of blood–brain barrier integrity may prevent cerebrovascular disease.

Transcortical electrical resistance (TEER) is used to assess the integrity of endothelial monolayers by measuring the electrical resistance of cell monolayers. The study placed mice bEnd.3 cells were treated in serum and urea from dialysis patients and showed a significant decrease in TEER (31), suggesting us CKD. results showed a significant decrease in TEER. Similarly immortalized human brain microvascular endothelial cells (ihBMEC) were subjected to the presence of uremic toxins and sodium fluorescein tracer showed increased permeability of ihBMEC (13). This further confirms the toxicity of uremic toxins on endothelial cells. However, there have been some reports of MRI imaging of the brain in patients with chronic kidney disease showing impaired BBB integrity (124). These macroscopic data provide important support for refining the mechanism of induction of CMBs after BBB injury in CKD patients. However, the ability of cerebral endothelial cells to phagocytose erythrocytes without damaging the monolayer of endothelial cells should be taken into account in recognizing “pseudo microbleeds” (125). The best approach is the assessment of endothelial cell integrity, which can be accomplished using vascular permeability marker assays (126).

It is well known that the blood–brain barrier prevents the entry of harmful substances into the brain, which can cause brain diseases. During the study of these harmful substances, we found that inorganic phosphate (Pi), indole sulfate (IS) and soluble receptor for advanced glycosylation end products (sRAGE) are toxic to brain endothelial cells (127–129). Tight junction proteins are proteins that safeguard the integrity of the BBB, and these toxins lead to a reduction in tight junction proteins and disruption of the actin cytoskeleton, which ultimately leads to impaired blood–brain barrier function, thus supporting the mechanistic role of uremic toxins in affecting BBB permeability and promoting CMBs.

It has been suggested that excess lipopolysaccharide (LPS) leading to neurological dysfunction (130, 131) is associated with blood–brain barrier disruption and brain DNA damage (13, 36, 132), in which microglia activation is thought to play an important role. Therefore, we conjecture that BBB disruption is also associated with microglia activation. Being found to abnormally activate the immune system and cause neuroinflammation, an animal model of adenine-induced senescence, the C57BL/6 J mouse, has now shown that CKD is able to induce a shift in microglia phenotype from quiescence to activation (13). This gives us a big hint that for altered body functions in CKD patients, disruption of the blood–brain barrier through activation of microglia can induce CMBs. the study was also validated by the PLX3397 diet, which revealed that a significant reduction in the number of microglia was followed by a significant reduction in the number of foci of CMBs (13), which strengthens the link between microglia and CMBs. The absence of hypertension in this animal model precludes the role of hypertension. However, the experiment was limited to old mice only. The prevalence of inflammation, increased oxidative stress, destruction of endothelial cells and even microglia destruction in old mice perhaps has a predisposing effect in itself. Future studies in which we can separately establish different age groups of mice to observe the phenomenon of CMBs and observe whether the same correlation exists will require further study. The increase in blood–brain barrier permeability caused by inflammation also triggers the entry of molecules such as IgG and promotes an increase in local barrier permeability, leading to erythrocyte leakage and toxic deposition. This links brain damage in chronic kidney disease to the accumulation of uremic toxins and disruption of the integrity of the blood–brain barrier, creating the “neurodegenerative” hypothesis (133).

In summary, disruption of the blood–brain barrier provides favorable conditions for the development of CKD-induced CMBs.

## 5 Treatment and prevention strategies

### 5.1 Progress and outcome assessment of treatment

Currently, more and more studies are focusing on the prevention and treatment of CMBs, including antihypertensive therapy, antiplatelet therapy, thrombolytic therapy, and statin therapy.

The management of hypertension in both CKD and CMBs is individualized, and if lifestyle interventions are poor, monotherapy or combination of medications should be used. Traditional drugs angiotensin converting enzyme inhibitors(ACEI), angiotensin receptor blockers (ARB) can control blood pressure well. A number of drug studies have been conducted in animal model experiments to attenuate CMBs associated with hypertension. Navitoclax, an anti-aging drug BCL-2 inhibitor, was able to induce apoptosis of senescent cells while protecting neurological function and reducing CMBs in hypertensive mice (56). Oral administration of pyrophosphate prevents vascular calcification in CKD Abcc6 mice (134), which has potential therapeutic value in vascular senescence due to CKD. However, although these findings are promising, they remain to be confirmed by clinical studies in humans.

Clinical studies have found a positive correlation between the use of anticoagulant drugs and the incidence of CMBs, especially warfarin (125). However, low molecular weight heparin is usually added to the blood during hemodialysis in patients with severe chronic kidney disease and is characterized by high bioavailability, safety, reduced bleeding tendency, and fewer complications. This suggests that there is still a complex relationship between anticoagulant use and CMBs. Our previous study concluded that patients with ischemic stroke combined with CMBs first need to control all risk factors for stroke, such as hypertension, diabetes mellitus, and atrial fibrillation (135). In patients with indications for anticoagulation and antiplatelets, relevant medications can be used routinely when the number of CMBs is less than 5; when the number of CMBs is 6–10, thrombolytic and antithrombotic therapies should be used cautiously with SWI monitoring; and antithrombotic medications should not be used when the number of microhemorrhagic foci exceeds 10 or more. Thus, although anticoagulants are essential in patients with comorbidities such as atrial fibrillation, their use in patients with chronic kidney disease who have CMBs requires careful risk–benefit assessment, especially given the elevated risk of bleeding. The latest renal guidelines state that thromboprophylaxis with non-vitamin K antagonist oral anticoagulants (NOAC) is preferred for patients with G1-G4 stage CKD (8). Telmisartan Effectively Prevents AngII-Induced Blood Pressure Elevation and Reduces AngII-Induced CMBs (136) Advocacy of glucose monitoring in CKD patients with cerebrovascular patients is said to be extremely important, and the guideline newly added sodium-glucose co-transporter protein 2 inhibitor (SGLT2i) as a first-line drug for the treatment of CKD (8). In particular, in patients with stage 5 CKD who are not yet on dialysis, the use of SGLT2i is associated with a lower risk of long-term dialysis (137).

This is effective in slowing down the progression of CKD and also in reducing the incidence of cerebral small vessel disease (138). Atorvastatin, as a representative of the statin class, is thought to reduce the incidence of CMBs through anti-inflammatory and anti-oxidative stress (139), its role in improving renal function has also been recognized, especially for patients with stage 3–4 CKD (140). However, Guidelines suggest that initiation of statin therapy is more risk-based and its more therapeutic effects still need to be explored (8). Activation of Nrf2-HO-1 signaling can control the oxidative stress process in CKD, and this treatment has been tested in animal models with promising results, but may fail in clinical trials. Antithrombotic drug therapy is considered clinically significant for disease control in the short term. For the risk of bleeding outweighs the benefit as the duration of treatment increases (141). In a limited longitudinal study, simvastatin 20 mg plus ezetimibe 10 mg daily was found to reduce LDL cholesterol and was effective in reducing thrombosis in patients with end-stage CKD (140). Therefore, the treatment of CKD-induced CMBs with this approach requires an ideal drug delivery system to minimize trial failures that may result from insufficient drug dosage (142).

### 5.2 Prevention strategies and prospects for clinical application

Controlling related risk factors is a relatively good preventive strategy. From the mechanism, we can find that for the strategy of preventing CMBs caused by CKD, we can start from the following aspects: (1) Closely monitor the degree of vascular calcification in CKD patients. Clinical prediction of cerebral hemorrhage by eGFR has been possible, mainly through intracranial arterial calcification as an intermediate biomarker. Closely monitoring the degree of intracranial calcification in CKD patients and actively implementing interventions are of great clinical significance in effectively avoiding the occurrence of cerebral microhemorrhage (90, 91). (2) Improvement of uremic toxins in CKD patients. Uremic toxins have a major role in inflammation and oxidative stress in promoting the mechanism of CMBs, and the use of probiotics, prebiotics, and a low-protein diet has been suggested to improve this gut imbalance as well as reduce gut barrier permeability, perhaps reducing the incidence of CMBs in CKD patients (121). (3) Targeting gut microbes. A large portion of gut imbalance is attributable to gut microbes, and targeting gut microbes may be a mechanism to attenuate or prevent the development of CMBs (143). (4) Giving supplemental vitamin D levels to CKD patients. Patients with CMBs are at risk for serum 25(OH)D deficiency, and clinical trials have demonstrated that vitamin D therapy has a beneficial effect on patients with end-stage renal disease by lowering inflammation-related markers, so serum 25(OH)D testing can also be performed when screening the CMB target population and appropriate supplementation can be administered when necessary (113). (5) Control common risk factors in CKD patients. For example, control of blood glucose, control of blood pressure, etc., in order to reduce the mechanisms associated with the induction of CMBs (Table 2).

## 6 Summary and outlook

This article focuses on the mechanisms by which chronic renal insufficiency contributes to the development of CMBs by affecting cerebrovascular autoregulation, inflammatory immune responses, blood–brain barrier disruption, and vitamin D deficiency. Chronic renal insufficiency promotes the release of inflammatory substances, the development of oxidative stress, and decreased compliance of cerebral small vessels due to excess calcium and phosphorus deposition, which are among the mechanisms we studied. Understanding the mechanisms by which chronic renal insufficiency induces CMBs could contribute to clinical prevention and further exploration of new mechanisms. Although our article suggests that chronic renal insufficiency is a potentially modifiable risk factor for CMBs, we need a large number of studies to explore how chronic renal insufficiency induces CMBs, such as whether targeted interventions for chronic renal insufficiency (e.g., inhibitors of the renin-angiotensin system, dietary protein restriction, etc.) reduce the progression of CMBs, and to assess the role of interventional therapies in protecting the Long-term benefits in cognitive function, such as the recently popularly researched brain-computer interface technology that can provide a good help in the rehabilitation of stroke patients (144). Combining this technology to further assess the extent of CMBs in CKD patients and to provide timely intervention may be a new field for the future. However, the existing animal models are mainly mice, and the establishment of animal models that are closer to the human brain and suitable for brain-computer interface experiments has great potential for its application. Meanwhile, the experimental period of the existing research animal models is relatively short, and future studies should focus on 10 years or more long-term clinical trials for evaluating targeted intervention therapies, which can more deeply analyze the mechanism of CKD-induced CMBs. In addition, whether CMBs, a commonly used biomarker of stroke, can also be used as an early biomarker of brain injury progression in patients with CKD is a direction that needs to be further explored (Table 3). Using multi-level validation such as clinical sample analysis, modeling was able to determine the sensitivity and specificity to strengthen the association. Completion of this work is a huge step forward in understanding mechanisms, exploring early prevention strategies, and screening for targeted agents that reduce the risk of morbidity.

**Table 3:** Future research directions for mechanisms related to CKD promoting the development of CMBs.

Knowledge gaps	Current limitations	Potential solutions
Does targeted intervention for chronic renal insufficiency reduce progression of CMBs	Lack of adequate research findings	Multi-omics data integration (renal metabolomics, brain imaging omics) may help reveal potential pathways.
Study of Vitamin D deficiency associated with inflammation and oxidative stress	Conflicting conclusions from different studies	Clarify the contradictory differences in conclusions, multi-dimensional experiments to verify the core pathway
Can CMBs serve as an early biomarker of brain injury progression in patients with chronic kidney disease	Lack of adequate research findings	Sensitivity and specificity were determined by multilevel validation (clinical sample analysis, mechanism exploration, modeling)
Evaluating the long-term benefits of intervention therapies in protecting cognitive functioning	Short follow-up period in existing clinical studies	Designing clinical trials for 10 years or more

## Glossary

CMBs: Cerebral Microbleeds
CKD: Chronic Kidney Disease
MRI: Magnetic Resonance Imaging
GFR: Glomerular Filtration Rate
CVD: Cerebrovascular Disease
ACR: Albumin Creatinine Ratio
eGFR: Estimated Glomerular Filtration Rate
ICH: Intracerebral Hemorrhage
BPV: Blood Pressure Variability
LPS: Lipopolysacchatide
HE: Hematoxylin–Eosin
DNA: DeoxyriboNucleic Acid
TNF-*α*: Tumor Necrosis Factor-α
IgG: Immunoglobulin G
BBB: Blood–brain barrier
TEER: Transepithelial Electrical Resistance
ihBMEC: immortalized human Brain Microvascular Endothelial Cells
ROS: Reactive Oxygen Species
MMP: Matrix Metalloproteinase
NOX: NADPH Oxidase
hsCRP: Hypersensitive C-reactive protein
TMAO: Trimethylamine N-oxide
IL-6: Interleukin-6
IFN-*γ*: Interferon-γ
NF-κB: Nuclear Factor kappa-light-chain-enhancer of activated B cells
Iba-1: Ionized Calcium Binding Adaptor Molecule 1
CK2: Casein Kinase 2
TGF-β1: Transforming Growth Factor beta
NADPH: Nicotinamide Adenine Dinucleotide Phosphate
MEK–ERK: Mitogen-activated extracellular signal-regulated kinase- extracellular regulated protein kinases
Pi: Inorganic phosphate
IS: Indoxyl sulfate
sRAGE: Solubility Receptor for Advanced Glycation End-products
eNOS: endothelial Nitric Oxide Synthase
PCS: P-Cresol Sulfate
PKC: Protein Kinase C
PI3K: Phosphoinositide 3-Kinase
SASP: Senescence-Associated Secretory Phenotype
TNFAIP3: Tumor Necrosis Factor Alpha-Induced Protein 3
IL-8: Interleukin-8
MCP-1: Monocyte Chemoattractant Protein-1
RAAS: Renin-Angiotensin-Aldosterone System
P: Phosphorus
FGF-23: Fibroblast growth factor-23
NRF2: Nuclearrespiratoty Factor 2
PBS: Phosphate buffer saline
t-BHP: Tert-Butyl Hydroperoxide
Nrf2-HO-1: Nuclear factor erythropoietin-2-related factor 2-Heme Oxygenase 1
IAC: Intracranial Calcification
